# Chicken ovarian follicles morphology and *growth differentiation factor 9* gene expression in chicken ovarian follicles: review

**DOI:** 10.1016/j.heliyon.2022.e08742

**Published:** 2022-01-11

**Authors:** V.R. Hlokoe, T.L. Tyasi, B. Gunya

**Affiliations:** Department of Agricultural Economics and Animal Production, University of Limpopo, Private Bag X1106, Sovenga, 0727, Limpopo, South Africa

**Keywords:** Preovulatory follicles, Pre-hierarchical follicles, *GDF9* gene, Morphology, Chickens

## Abstract

Ovary follicular development is a progressive system from the beginning of small cortical follicles to the ovulation of hierarchical follicles. The review was conducted to provide information on the indigenous chickens commonly used for egg production, chicken ovarian follicles morphology and expression of *growth differentiation factor 9* (*GDF9*) gene in ovarian follicles and its relationship with egg production. The research databases used in the study include google scholar, Science Direct, PubMed, JSTOR and Cambridge Core. Google, Yahoo and Baidu search engines were used to search the information. In this study, the papers selected for use were original research articles and reviews to ensure that the information used was from research results. Besides, only recent English papers, 2010–2021, were used. The keywords used to search for articles were chicken ovarian follicles, ovarian morphology and *GDF9* gene expression. The documents showed that pre-hierarchical follicles include many small and large white follicles, which are about 2–5mm in diameter and 5 to 6 small yellow follicles (SYF) that are about 5–10mm in diameter. Preovulatory follicles are about five to six in number and above 10mm in diameter, with the sizes from F6 to F1, with F1 as the largest follicle. Further, the studies revealed that *GDF9* gene mRNA is expressed in the highest concentration in small yellow follicles and other studies reported that the expression of *GDF9* gene has been found in follicles of the primary to preovulatory stages in chickens. This review concludes that the *GDF9* gene expression is mainly throughout follicular growth and it stimulates the proliferation of pre-hierarchical granulosa cells. The increased egg production in chickens depends on progressive developmental stages and the growth of ovarian follicles.

## 1 Introduction

In the worldwide poultry industry, egg-laying production efficiency is a significant economic trait. Excellent productivity is measured by the number of ovarian follicles heading for ovulation or atresia and the efficiency of the oviduct to convert the ova into a hard-shelled egg. A follicular hierarchy that is well-organised is important for improved egg laying performance. Yang et al. (2019) and Mfoundou et al. (2021) showed that egg production of geese is related to the follicular development. *Growth differentiation factor 9* (*GDF9*) is one of the transforming growth factor-b (TGF-b) superfamily and plays an exclusive role in female fertility (Huang et al., 2015). *GDF9* genes have been found to have an effect in controlling chicken ovarian follicular growth and ovulation, with a great influence in laying hens. *GDF9* gene is one factor secreted by the oocyte with an important role in regulating ovarian function in female reproduction, modifying both the cell fate of the somatic granulosa cells and the quality and growing ability of the egg (Lou et al., 2018). Also, it is essential in controlling follicular physiological roles and is a major gene for regulating reproductive traits in different species. According to Li et al. (2019) *GDF9* might control the basal levels excretion by granulosa cells, though the influence varies in numerous animals. Due to the increasing human population, there is a high demand for eggs, hence, there is a need to evaluate the chicken ovarian follicles morphology, development and the expression of the *GDF9* gene in the follicles since it plays a huge role in the development of the follicles (Yang et al., 2019). The laying performance of chickens is determined by the growth, development and function of chicken ovarian follicles (Johnson, 2015). The laying of eggs starts with an orderly development of follicles in the chicken ovaries until the eggs are produced. Therefore, without the progressive stages of ovarian follicles’ growth and development, the chickens' egg production performance will be reduced (Wang et al., 2017; Li et al., 2019).

Several studies reported that improvement of the chicken ovarian follicles improved the egg production traits of the Chinese dagu chicken genotype (Zhang et al., 2012), Nigerian local chicken genotype (Gwaza et al., 2016) and Egyptian Alexandria chicken genotype (Soliman et al., 2020). Studies have been conducted to determine the role of the *GDF9* gene in chicken ovarian follicles (Otsuka et al., 2011; McDerment et al., 2012; Qin et al., 2015) and the expression of the *GDF9* gene in chicken ovarian follicles of Chinese local chickens (Huang et al., 2015) and Single-comb White Leghorn hens (Johnson et al., 2005). However, we say this under correction, there is no study summarising the findings of chicken ovarian follicles morphology and the expression of the *GDF9* gene in the chicken ovarian follicles. Hence, the objectives of the review were to provide information on the chicken ovarian follicles morphology and the *GDF9* gene expression in chicken ovarian follicles.

The literature review of the study will be focusing on the topics, some of the indigenous egg-laying chicken genotypes (Potchefstroom Koekoek, Venda, Botchveld and Ovambo), morphological characterisation of chicken ovarian follicles and *GDF9* gene and its association with the chicken egg production.

## 2 Materials and methods

The study used literature review to produce research discoveries and it covers areas where additional research is required. Several research outcomes that have a relation with local chickens and some associated issues were reviewed, shown and referenced. Reports of studies that focused on chicken ovarian follicles morphology and *GDF9* gene expression levels in chicken ovarian follicles were reviewed as well. Firstly, research databases were identified and selected for searching the information, which included google scholar, Science Direct, PubMed, JSTOR and Cambridge Core. Google, Yahoo and Baidu search engines were used. Secondly, the keywords including chicken ovarian follicles, ovarian morphology and *GDF9* gene expression were used for article searches. Thirdly, the papers selected for the study were original research articles and reviews to ensure that the information used was from research results. Only recent English papers, 2010-2-21, were used to ensure the results are still relevant to the current conditions. The research found 31,700 papers using keyword chicken ovarian follicles, 77,124 using ovarian morphology and 11 500 using *GDF9* gene expression. The papers with the languages that could not be understood by the authors were excluded. Studies were considered for inclusion in the review provided, they dealt with chicken ovarian follicles morphology, *GDF9* gene expression in chicken ovarian follicles and its association with production of eggs as well as studies of chicken egg-laying. Research outcomes of egg production of indigenous chickens were also considered for inclusion in some sections of the review. In this way, the results were narrowed and, in the study, 44 papers were used and cited.

### 2.1 Some of the indigenous egg-laying chicken genotypes

#### 2.1.1 Potchefstroom Koekoek chicken breed

**Figure 1:**
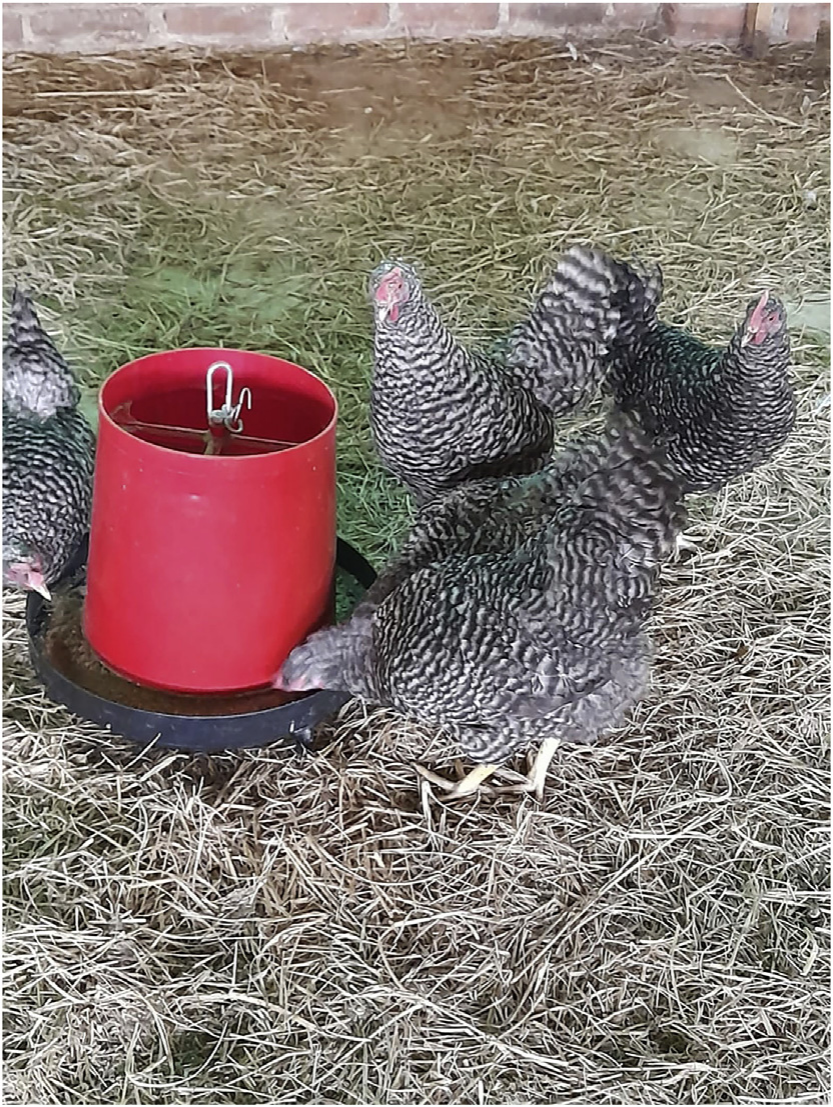
Potchefstroom Koekoek breed.

The Potchefstroom Koekoek (Figure 1) is the chicken genotype that was produced by the researcher, Marais, in the 1950s at Potchefstroom Agricultural College (Dessie and Gatachew, 2016). According to Tyasi et al. (2019), Potchefstroom Koekoek is an indigenous chicken genotype, which was formed through the crossing of Black Australorp, Bared Plymouth Rock and White Leghorn. The word “Koekoek” is referring to the colour patterns of the genotype. The colouring of the feathers is sex-linked, making it useful during the breeding programme. The chicken type is well adapted to the tropical regions and that enables it to survive from the hot climatic conditions and is a dual-purpose chicken genotype best suited for free-range farming operations (Dessie and Gatachew, 2016). The chicken genotype can sustain itself, is disease resistant, has excellent temperament and it was developed for traits such as egg production and carcass with attractive yellow skin colour (Mutibvu et al., 2019). Besides, the genotype is having black and white stripped feathers and yellow legs as shown in Figure 2.01 and the hens are broody and make for good sitters (Magothe et al., 2012). On average, the hens can produce about 198 eggs per annum and the average weight of the eggs is about 55.78 g (Mtileni et al., 2012). The hens can lay large eggs with brown shells which have rich yellow to orange yolks (Heit, 2017). It is a very popular genotype among South African rural farmers and neighbouring countries for meat and egg production (Mtileni et al., 2012).

#### 2.1.2 Venda chicken breed

**Figure 2:**
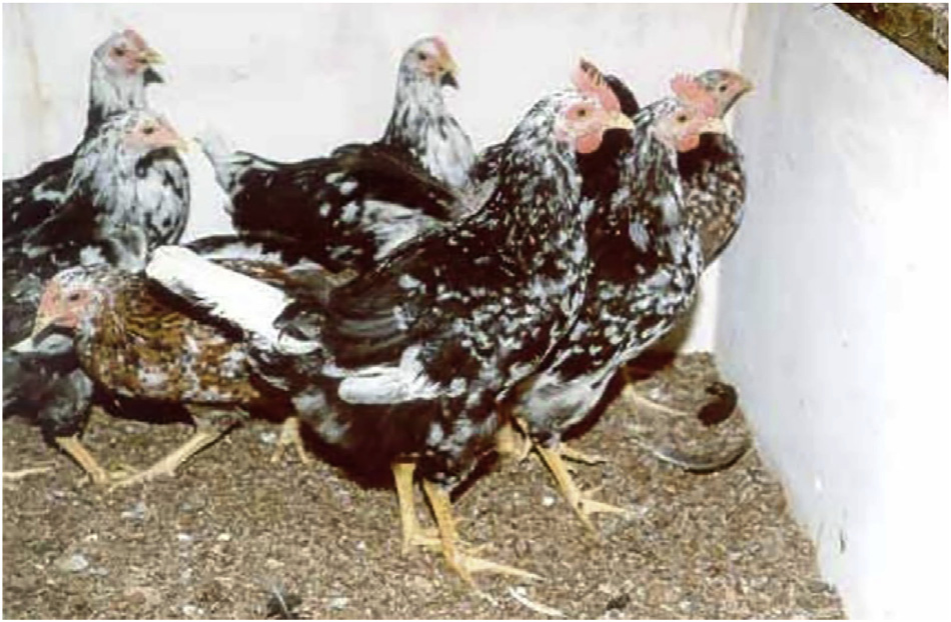
Venda chicken breed.

The description of Venda chicken was first done by the veterinarian, Dr Naas Coetzee, and was named after the previous Venda province that is now part of the Limpopo province in South Africa (Norris and Ngambi, 2006). Venda chickens have multi-coloured feathers with black, white and red as predominating colours, rose-coloured combs and five-toed feet are common (Figure 2). The genotype is large and can lay large, tinted eggs with an average of 70 eggs per annum and an average weight of 53 g (Mtileni et al., 2011). Venda chicken genotype has good mothering ability, broodiness, high survivability, can survive harsh environmental conditions and is resistant to diseases (Ngambi et al., 2013). They are good scavengers and can sustain themselves well without feed provision. They feed on many diets including seeds, household leftovers, insects, lizards and small rodents (Mabelebele et al., 2014).

#### 2.1.3 Boschveld chicken breed

**Figure 3:**
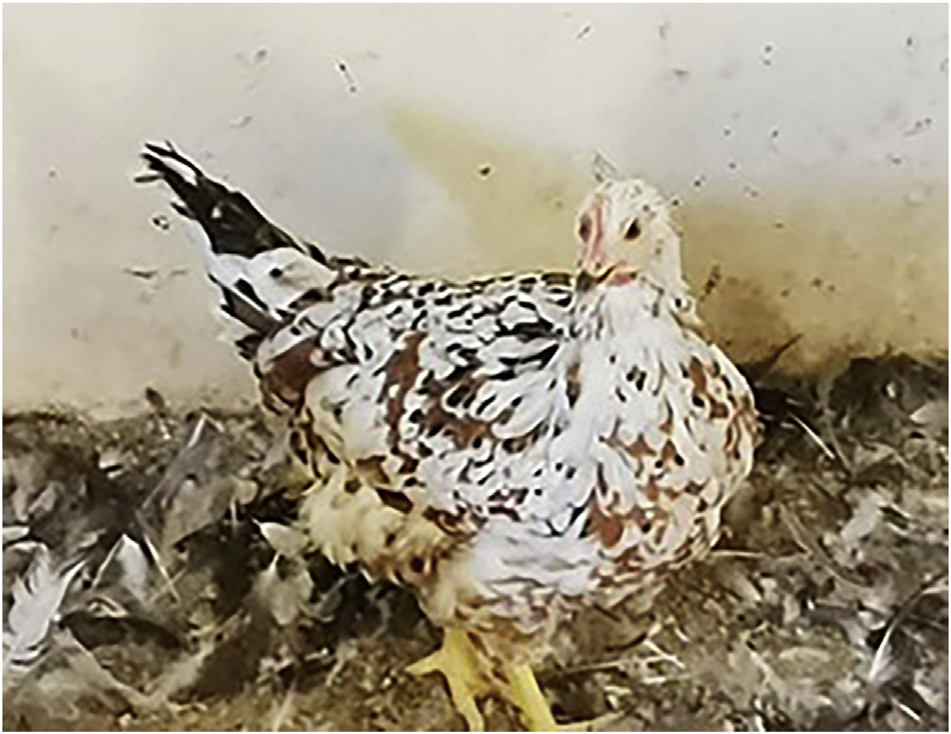
Boschveld chicken breed.

According to Bosch (2011), Boschveld (Figure 3) is an indigenous chicken genotype that was developed through the crossing of three native genotypes namely Venda, Matabele and Ovambo. The genotype has good self-sustainment, can move around searching for food for survival and is well adapted to extreme environmental surroundings. Dessie et al. (2011) reported that the Boschveld chicken genotype was bred to be resistant against diseases, for faster growth and it can perform well on a free-range system with homemade rations and under scavenging conditions. The chicken genotype is large, hardy, has a mixture of brown and white feathers. The hens are good brooders with a great mothering nature and can reproduce well in harsh environments. The hens can lay medium brown shelled eggs and an average of 200 eggs per annum.

#### 2.1.4 Ovambo chicken breed

**Figure 4:**
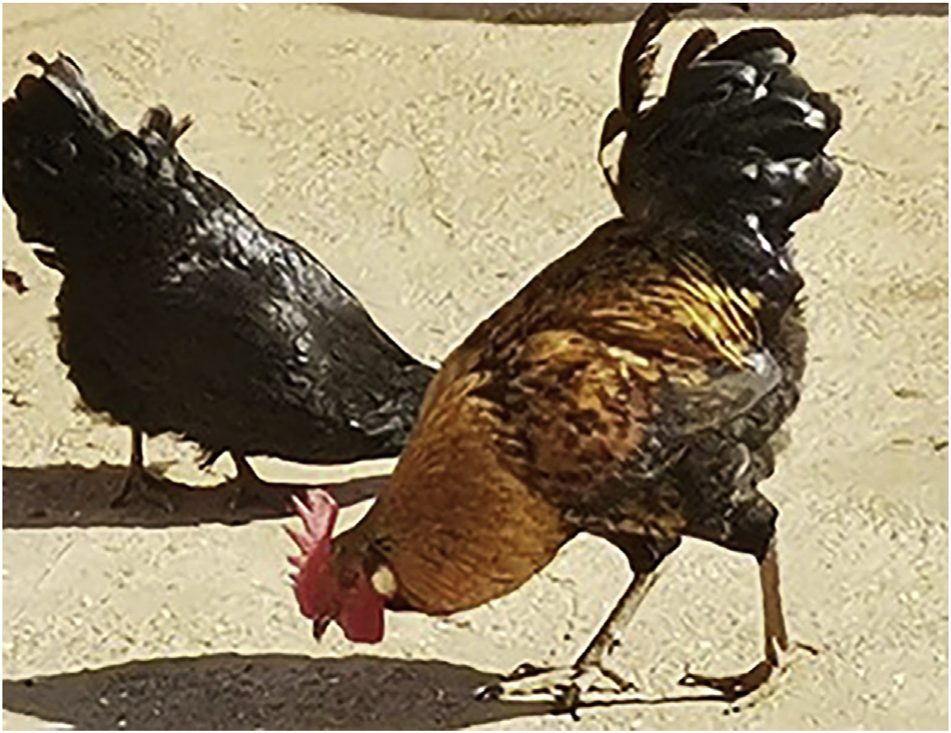
Ovambo chicken breed.

The Ovambo chicken genotypes are from the Northern part of Namibia and Ovamboland. They can perform well under a low management system (Bett et al., 2013). Ovambo chicken genotype (Figure 4) is a small chicken genotype with different colour patterns, which assists them to camouflage to protect themselves against predators. Their small size permits them to fly and roost on top of the trees to escape the predators. The ovambo chicken breed has feathers of a dark to black colour, with stripes of white and or orange (Grobbelaar et al., 2010). These chicken genotypes are characterised as layers and can survive well under harsh conditions. Their body weights average 1.32 kg at 16 weeks and 1.54 kg at 20 weeks (Bett et al., 2013).

### 2.2 Morphological characterization of chicken ovarian follicles

**Figure 5:**
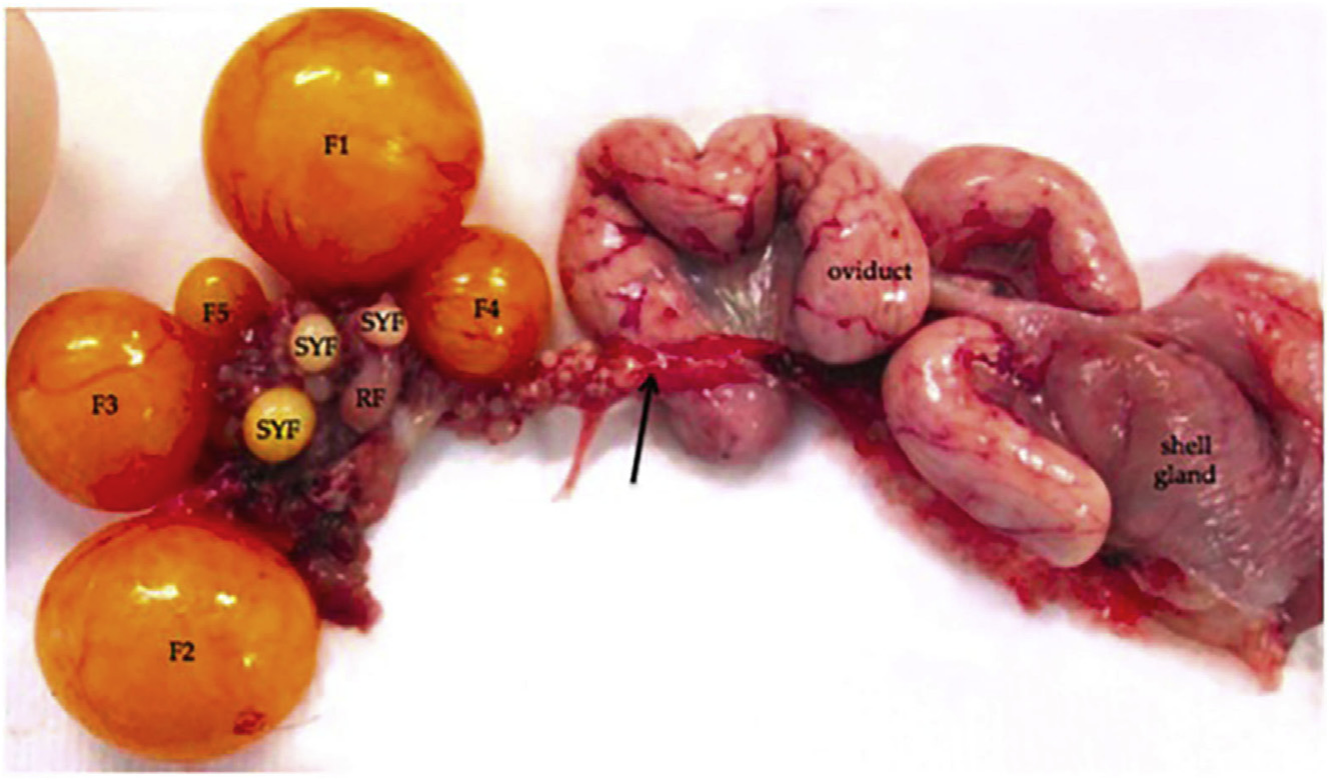
Chicken ovarian follicles (Apperson et al., 2017). Five preovulatory follicles are present (F1–F5). Small yellow follicles (SYF) and a degenerating post-ovulatory follicle (RF) are visible. The black arrow symbols nerves and blood vessels. The oviduct and shell gland are labelled.

The chickens' egg production performance depends largely on the progressive phases of growth and development of ovarian follicles categoried into 2 classes viz. pre-hierarchical and hierarchical follicles (preovulatory follicles) (Wang et al., 2017). Folliculogenesis is the process of the development of ovarian follicles from primordial follicles to a well-developed follicular hierarchy, which includes growth and multiplication of cells and differentiation before ovulation or follicle deterioration (Yu et al., 2016). The pre-hierarchical follicles include many small and large white follicles, which are about 2–5mm in diameter and 5 to 6 small yellow follicles (SYF), which are about 5–10mm in diameter. Hierarchical follicles, also known as preovulatory follicles, are about 5–6 in number and above 10mm in diameter (Wang et al., 2017). The ovary of layers consists of an order of yellow yolky follicles, known as the preovulatory follicles from F5 to F1 (Figure 5) and many thousands of small follicles from which large yolky follicles are conscripted (Apperson et al., 2017). Johnson (2014) reported that follicles develop from a category of small yellow follicles into the preovulatory hierarchy approximately once a day during the laying period, with a process called follicle selection. The chosen follicle will develop faster from an F6 follicle to an F1 follicle until ovulation occurs. According to Wang et al. (2014), the preovulatory ovarian follicles develop in order from F6 to F1, with F1 as the largest follicle, which is ready to be ovulated next and F2 as the second largest follicle followed by others until F6 as the smallest preovulatory follicle. According to Yang et al. (2019), Yangzhou geese follicle numbers are more than that in Zhejiang geese and Carlos geese in the egg-laying periods. The differences in the number and weight of follicles among the three breeds were observed and this might be associated with egg production. Zhang et al. (2015) established that commercial Hy-line hens had more hierarchical follicles and bigger ovary weight than in Chinese indigenous hens. It was stated that the proliferation of the granulosa cells promoted pre-hierarchical follicles to enter hierarchical growth in geese, meaning that the thicker the granulosa cell layer, the more pre-hierarchical follicles get mature and the more egg production (Yang et al., 2019). The expression levels of the genes vary at different stages of follicle development, hence, it is important to highlight the expression of the *GDF9* gene at different stages of follicles (Pablo et al., 2018).

### 2.3 *Growth differentiation factor 9* gene and its association with egg production in chickens

*GDF9* gene is interestingly appearing in the oocytes and granulosa cells of ovarian follicles in chickens and is a central controller of folliculogenesis and ovulation rate (Otsuka et al., 2011). According to McDerment et al. (2012), the *GDF9* gene has been found to play a significant role in the development and maturation of ovarian follicles in chickens, with a possible influence on the production of eggs in laying chickens. According to Huang et al. (2015), the relationship between numerous *GDF9* SNPs and egg production traits in Chinese indigenous chicken types highlighted the essential role of *GDF9* in the growth of hen ovaries. The literature on chicken ovaries has revealed that the expression of *GDF9* is mainly throughout follicular growth and it has an influence on the proliferation of pre-hierarchical granulosa cells (Qin et al., 2015). Besides controlling folliculogenesis, GDF9 also regulates the other genes that are expressed in granulosa cells. The Gremlin protein had its expression improved by *GDF9* in murine granulosa cells as well as INHB and steroidogenesis acute controlling protein, which also had improved expression whereas INHA had its expression reduced by *GDF9* (Ernst et al., 2018; Sanfins et al., 2018). The *GDF9* expression pattern was closely associated with ovarian growth in *Schizothorax prenanti* (fish). Compared with other non-gonadal tissues, the ovary showed more expression of the *GDF9* mRNA. Although the *GDF9* mRNA is expressed at high levels in the ovary, its expression shows a stage-specific pattern during ovarian development, and it is expressed at greater levels during the start of primary oocytes from oogonium or vitellogenesis during oocyte maturation (Yan et al., 2020). It was also reported that lack of *GDF9* expression in mice affected follicle growth and led to infertility. *GDF9* can decrease the biological effects of FSH in undifferentiated granulosa cells, control the development of granulosa cells and constrain the premature luteinisation of granulosa cells (Lou et al., 2018). According to Johnson et al. (2005), *GDF9* gene mRNA is expressed in the highest concentration in small yellow follicles in Single-comb White Leghorn hens. Another study reported that the expression of the *GDF9* gene has been found in follicles of the primary to preovulatory stages in chickens (Hayashi et al., 2009) and mice (Lou et al., 2018). According to Wang et al. (2013), the normal expression of the *GDF9* gene permits the down regulation of *inhibin A*, thus, encourages the capacity of the follicles to progress through the primary stage of growth. Besides, it stimulates the growth of preantral follicles by stopping granulosa cell apoptosis. In studies including the shutdown of *GDF9* expression in mammals, oocytes displayed irregular development and with the deactivation of *GDF9*, folliculogenesis got disturbed in the primary stage of growth, leading to the non-formation of mature follicles, ovulations and, subsequently, pregnancies (Castro et al., 2016; Sanfins et al., 2018).

## 3 Conclusion

The current literature review was conducted to investigate the literature on the expression levels of the *GDF9* gene in ovarian follicles of chickens. The literature review focused on some of the indigenous egg-laying chicken genotypes (Potchefstroom Koekoek, Venda, Boschveld and Ovambo), morphological characterisation of chicken ovarian follicles and *growth differentiation factor 9* gene and its association with chicken egg production. The review displayed that the increased egg production in chickens depends on progressive developmental stages and the growth of ovarian follicles. *GDF9* gene has shown to play an essential role in folliculogenesis and granulosa cells proliferation in chickens, which leads to improved follicle development and that a lack of *GDF9* expression might affect the follicle growth and lead to infertility. However, not much work has been conducted on the expression levels of the *growth differentiation factor 9* gene in chicken ovarian follicles. Therefore, there is a need for investigating the expression levels of the *GDF9* gene in ovarian follicles of chickens.

## Declarations

### Author contribution statement

All authors listed have significantly contributed to the development and the writing of this article.

### Funding statement

This research did not receive any specific grant from funding agencies in the public, commercial, or not-for-profit sectors.

### Data availability statement

No data was used for the research described in the article.

### Declaration of interests statement

The authors declare no conflict of interest.

### Additional information

No additional information is available for this paper.
